# A Bacterial Analysis Platform: An Integrated System for Analysing Bacterial Whole Genome Sequencing Data for Clinical Diagnostics and Surveillance

**DOI:** 10.1371/journal.pone.0157718

**Published:** 2016-06-21

**Authors:** Martin Christen Frølund Thomsen, Johanne Ahrenfeldt, Jose Luis Bellod Cisneros, Vanessa Jurtz, Mette Voldby Larsen, Henrik Hasman, Frank Møller Aarestrup, Ole Lund

**Affiliations:** 1 Department of Systems Biology, Technical University of Denmark, Kemitorvet Building 208, 2800 Kgs. Lyngby, Denmark; 2 National Food Institute, Technical University of Denmark, Søltofts Plads Building 221, 2800 Kgs. Lyngby, Denmark; Tianjin University, CHINA

## Abstract

Recent advances in whole genome sequencing have made the technology available for routine use in microbiological laboratories. However, a major obstacle for using this technology is the availability of simple and automatic bioinformatics tools. Based on previously published and already available web-based tools we developed a single pipeline for batch uploading of whole genome sequencing data from multiple bacterial isolates. The pipeline will automatically identify the bacterial species and, if applicable, assemble the genome, identify the multilocus sequence type, plasmids, virulence genes and antimicrobial resistance genes. A short printable report for each sample will be provided and an Excel spreadsheet containing all the metadata and a summary of the results for all submitted samples can be downloaded. The pipeline was benchmarked using datasets previously used to test the individual services. The reported results enable a rapid overview of the major results, and comparing that to the previously found results showed that the platform is reliable and able to correctly predict the species and find most of the expected genes automatically. In conclusion, a combined bioinformatics platform was developed and made publicly available, providing easy-to-use automated analysis of bacterial whole genome sequencing data. The platform may be of immediate relevance as a guide for investigators using whole genome sequencing for clinical diagnostics and surveillance. The platform is freely available at: https://cge.cbs.dtu.dk/services/CGEpipeline-1.1 and it is the intention that it will continue to be expanded with new features as these become available.

## Introduction

Rapid and reliable identification, characterisation and comparison of microorganisms from food, human, animal and environmental samples are essential to guide clinical treatment, infectious disease control and to detect outbreaks. Today, bacterial diagnostics are typically performed using a variety of different phenotypic and increasingly genotypic methods [1]. Recent developments in microbial whole genome sequencing (WGS) hold great promise for enhancing diagnostics and public health microbiology [2–4]. The great value of WGS in studying bacterial evolution, outbreaks and transmission has been shown in a number of recent studies [5–8].

The next step is to translate this technology from a research tool into one with utility in routine diagnostic settings. Retrospective or real-time use of benchtop sequencing for selected isolates has indicated the great potential of the technology for understanding and potentially limiting intrahospital transmission of these selected pathogens [9–11]. Similarly, for food-borne diseases it has been shown that it is feasible to sequence *Salmonella* isolates in real-time and share the data to rapidly identify and potentially control outbreaks [12].

A major obstacle for the global implementation of WGS in routine diagnostic laboratories worldwide is the lack of rapid, easy-to-use, reliable and harmonised bioinformatics tools for storing, analysing and comparing data. Over the past couple of years, several online individual typing tools have been developed amongst others as part of the Center for Genomic Epidemiology (CGE) project for analysing relevant clinical features in WGS data. These include species identification[13], multilocus sequence typing (MLST) [14], detection of transferrable antimicrobial resistance genes[15], detection of plasmids in *Enterobacteriaceae*[16], prediction of pathogenicity[17], detection of virulence factors in *Escherichia coli*[5] and determination of phylogeny by Single Nucleotide Polymorphisms [5,18,19].

Here, we present a new web-based platform where several of the services mentioned above are integrated in a single, static and robust workflow. In addition, the platform contains several important features that the users of the services have requested [20]. First of all, the platform provides a default bacterium analysis pipeline that is executed on all uploaded samples. If required, the pipeline performs a draft de novo assembly of the sequencing reads into contigs, and continues to analyse the taxonomy, plasmid profile, resistance profile and virulence profile. Secondly, the platform provides a batch upload system where users can provide metadata for all their samples in a standardised Excel spreadsheet and upload this together with the corresponding WGS files in a single process. Finally, the platform provides a user login feature, which enables the users to store their data and rerun the provided services using their previously uploaded data. This feature grants the user full access to view and interact with their data. To the best of our knowledge no similar platform exists to compare against, but in this study we have benchmarked the performance of the individual services in the pipeline.

## Material and Methods

Datasets from a number of previous studies [5,8,14,21] were used to benchmark the BAP. This enabled the comparison of the results from the pipeline to those previously obtained using individual services. The combined dataset contained samples from the following species: 50 *Enterococcus faecalis* fasta files, 46 *Enterococcus faecium* fasta files, 43 *E*. *coli* fastq files, 49 *E*. *coli* fasta files, 4 *Klebsiella pneumoniae* fastq files, 50 *Salmonella enterica* fasta files and 93 *Staphylococcus aureus* fastq files. The 43 *E*. *coli* fastq files were Ion Torrent, while the remaining fastq files were Illumina data. For the MLST benchmark data, the expected MLST was in most cases inferred from the spa type, and thus only the clonal complex is available for these samples[21].

### Technical Machine Specs

The CGE platform is running on three machines, one web server and two compute servers with the following specs respectively: one Intel® Xeon® Processor X5660 with 24 cores and 96GB RAM and two Intel® Xeon® Processor E5-2690 with 32 cores and 384GB RAM each. The machines are all running Linux with openSUSE.

### The Bacterium Analysis Pipeline

**Fig 1:**
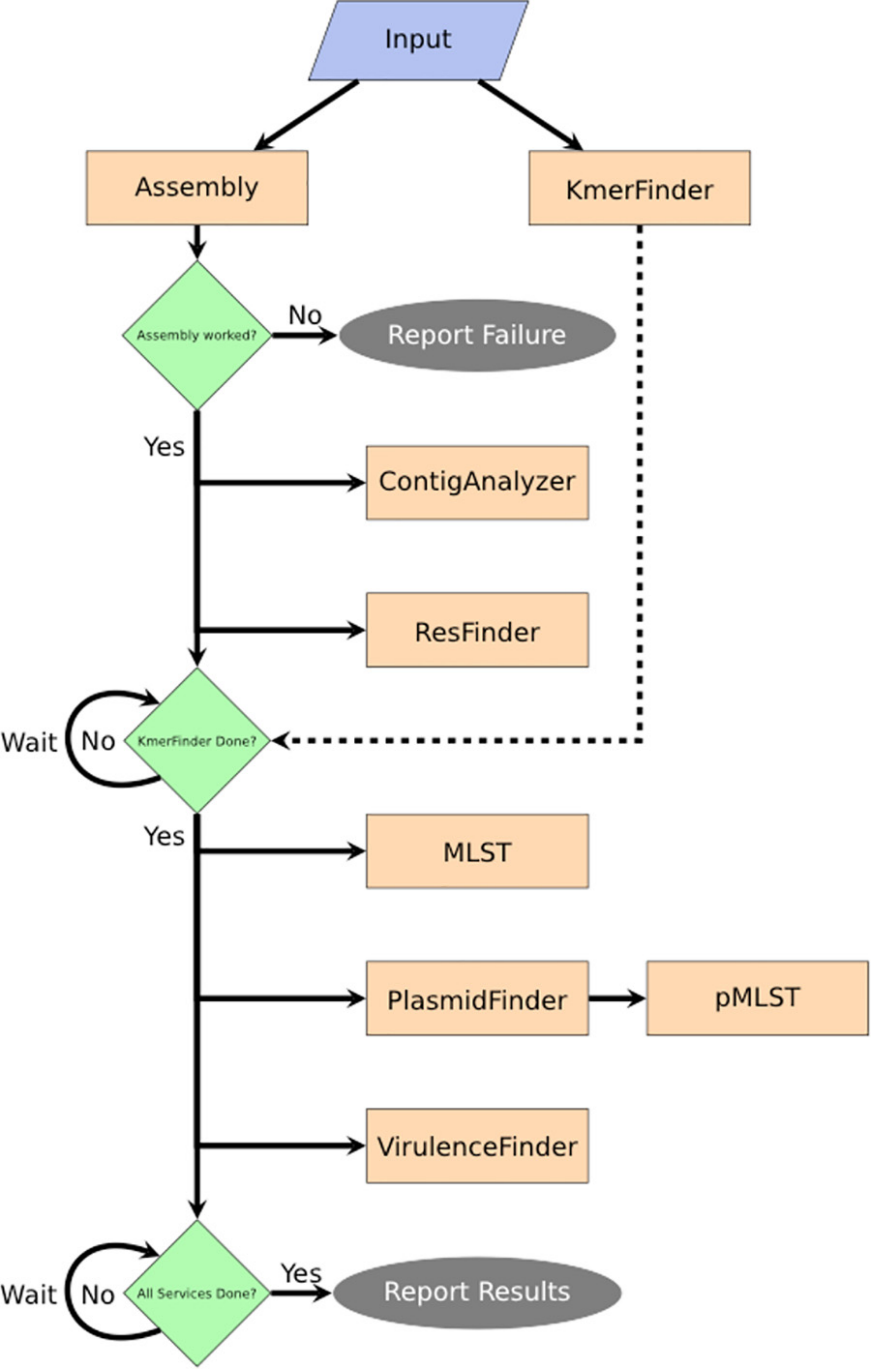
Flowchart depicting the workflow of the BAP. The input from the user is assembled if needed, and the bacterial species is identified through the KmerFinder algorithm. When ready, the assembled contigs are submitted to the ContigAnalyzer and ResFinder for annotation of contig metrics and identification of resistance genes. If the bacterial species is identified, the contigs are further, if applicable, submitted to MLST, PlasmidFinder and VirulenceFinder to identify the sequence type, known plasmids (and, if applicable, their plasmid sequence type), and known virulence genes. When all services are done, the BAP produces a summary report of the services result (see Fig 2).

**Fig 2:**
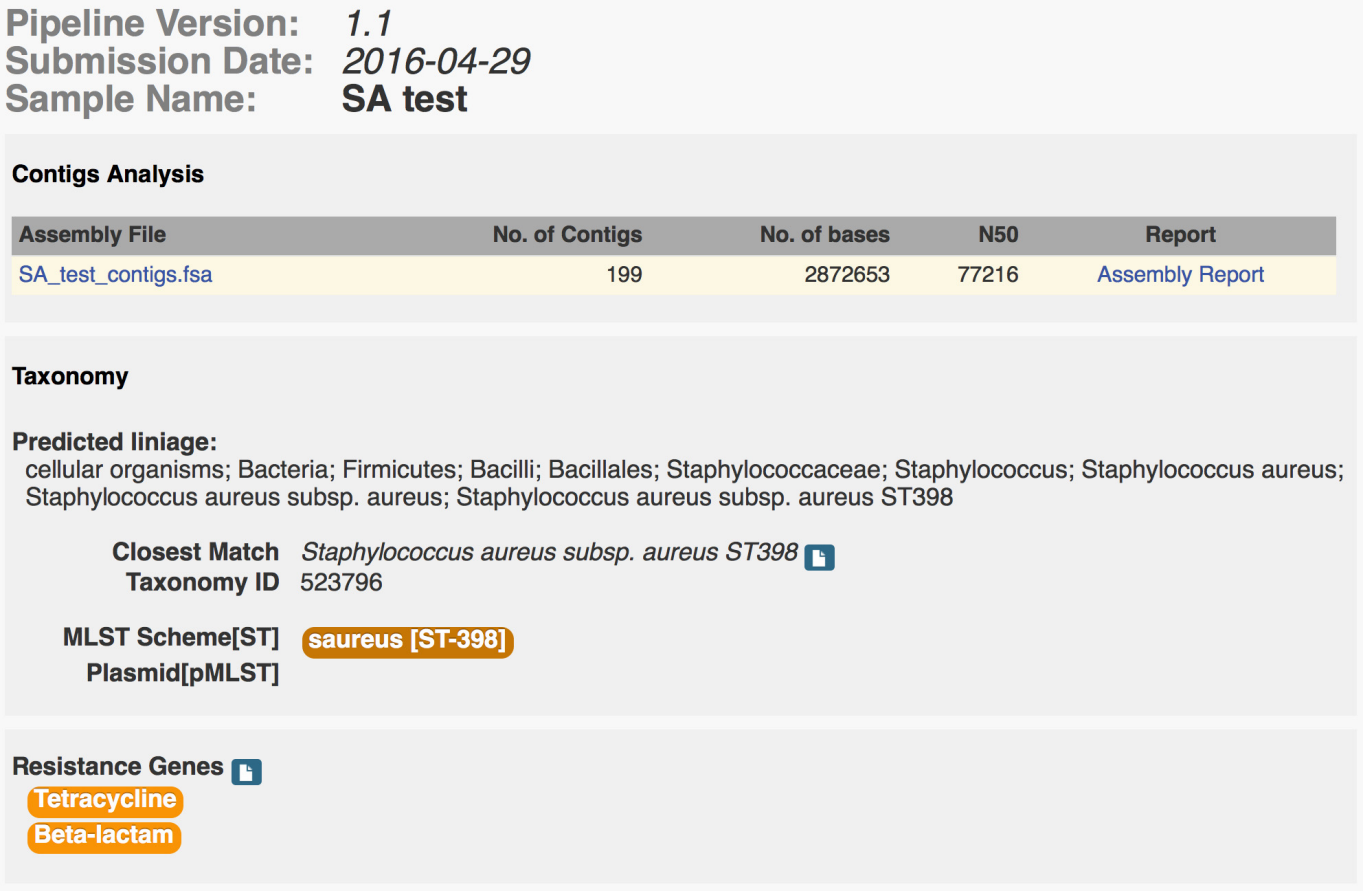
Example of a BAP summary report for a *S*. *aureus* sample. The report is split in 3 parts: Contigs/assembly analysis, taxonomy analyses and phenotypic analyses. Only the major result details are shown here, but the user is provided direct links to see the full report from the individual services.

An automatic and robust tool for analysing bacterial genomes, the Bacterium Analysis Pipeline (BAP), was developed to control the workflow, and to facilitate inter-program communication and user feedback regarding the status of the submitted jobs. The BAP combines several previously created services to produce a comprehensive single bacterial isolate analysis. The BAP (see Fig 1) starts out by assembling the submitted sequence reads using the methodology described earlier[14] and running a k-mer-based species identification algorithm previously shown to be highly accurate (KmerFinder [13,22]). These two services run in parallel. After the assembly into contigs, the BAP executes a contig analysis script and an acquired antimicrobial resistance gene identification service (ResFinder[15]). When the identification of the bacterial species is completed, the BAP continues to execute the remaining services: a multilocus sequence typing service (MLST[14]), a plasmid identification service (PlasmidFinder[16]), a plasmid sequence typing service (pMLST[16]) and a virulence gene identification service (VirulenceFinder[5]). These services, however, are only run if a relevant database exists for the identified species, and in the case of MLST a scheme for the given bacteria. Execution of pMLST further depends on whether a plasmid-subtyping scheme exists for the plasmid replicons identified by PlasmidFinder. When the BAP terminates successfully it will produce an interactive on-screen report summarising the major results from the executed services (see Fig 2). It is possible to receive an email upon finished analysis if the user provides an email.

### The BAP Services

The BAP uses our own internal assembly pipeline. The assembly pipeline has been evaluated using Illumina and Ion Torrent fastq files. The pipeline may handle fastq files from other sequencing platforms, but we recommend in those cases that the user do the assembly locally and upload the assemblies instead. Also note that our assembler only accepts DNA sequences in fastq format, however these fastq files may be zipped or gzipped. The pipeline uses the Newbler algorithm for Ion Torrent data, and velvet *de novo* for Illumina sequencing data. The Assembler generates a fasta file containing the contigs. For more details see Larsen et al. 2012 [14]. To annotate the contigs with some trivial, but useful, metrics we use a small in-house service, ContigAnalyzer, to calculate the number of contigs, total number of bases, the N50, the length of longest and shortest contig, and the median, mean and standard deviation of the contigs length.

The BAP uses the KmerFinder algorithm to predict the species of the bacteria samples. This algorithm uses DNA sequences provided in either fasta or fastq format. The k-mer size used by KmerFinder for bacterial data is 16 bases. The database is constructed from a monthly updated extraction of whole bacteria genomes from the National Center for Biotechnology Information, and only contains genomes that have registered taxonomy associated. KmerFinder produces a summary table of all significant hits. The pipeline only accepts the species to be the same as the closest match if the template coverage is above 50 per cent. If not the sample is annotated as an unknown species. More information on the KmerFinder service can be found in the original manuscripts [11,13].

The BAP uses the ResFinder algorithm to identify acquired antimicrobial resistance genes. The algorithm takes a fasta file containing DNA sequences as input. The algorithm uses BLAST[23] to find DNA sequences from the input sample, which matches the known resistance genes in the database. The database is manually curated and contains genes associated with the following resistance phenotypes: Aminoglycoside, Beta-lactam, Fluoroquinolone, Fosfomycin, Fusidic Acid, Glycopeptide, MLS—Macrolide-Lincosamide-StreptograminB, Nitroimidazole, Oxazolidinone, Phenicol, Rifampicin, Sulphonamide, Tetracycline and Trimethoprim. Note that this algorithm only finds genes contained in the database, and will as such not identify potentially novel genes nor chromosomal mutations associated with antimicrobial resistance. The algorithm provides an overview table of the found matches in the database, with the identity and the coverage of the hit, and the position of the gene in the input file. For additional details see Zankari et al. 2012 [15].

To identify the multilocus sequence type for the sample, when a MLST scheme exists for the given species, the BAP uses the MLST algorithm. Similar to ResFinder, the MLST algorithm uses the BLAST algorithm to find DNA sequences from the input sample that match the set of household genes found in the given MLST scheme database. If all genes have perfect matches then the MLST algorithm is able to use the found alleles to predict the MLST type. The database consists of fasta files, is updated weekly from the PubMLST.org site and currently contains 117 different MLST schemes (25 Aug, 2015). The MLST algorithm produces an overview table of the best matches for each of the 7 genes and annotates the identity, coverage and position similarly to ResFinder. See Larsen et al. 2012 for more information [14].

The BAP uses the PlasmidFinder algorithm to identify the plasmids contained in the input sample. The algorithm works similarly to ResFinder and uses the same input type, but it uses a different database containing plasmids. The database currently contains 117 replicon marker sequences of plasmids for species belonging to *Enterobacteriaceae*, and is manually curated. The algorithm provides an overview of the identified plasmids similar to ResFinder. For more details see Carattoli et al. 2014 [16].

To identify the plasmids multilocus sequence type in the input sample, the BAP uses the pMLST algorithm. The algorithm works similarly to MLST and uses the same input type, but it uses a different database containing plasmid schemes. The database currently has a scheme for plasmids belonging to IncF, IncHI1, IncHI2, IncI1 and IncN, and is manually curated. The pMLST algorithm produces an overview table of the best matches for each of the genes in the pMLST scheme, and annotates the identity, coverage and position similarly to MLST. For additional information see the original manuscript [16].

The BAP uses the VirulenceFinder algorithm to identify the virulence genes contained in the input sample. The algorithm works similarly to ResFinder and uses the same input type, but it uses a different database containing published virulence genes. Currently the database only has virulence genes for the species *E*. *coli*. The database is manually curated. The algorithm provides an overview of the identified virulence genes similar to ResFinder. See Joensen et al. for more details [5].

### The Batch Upload System

The web interface of the Batch Upload System provides a possibility for the user to upload WGS data from several samples in a single submission, which significantly enhances the usability of the BAP, as most laboratories will have more than one sample to analyse at the same time. The interface constitutes only one of the three parts of the Batch Upload System. The other two parts are a file assembly script and a batch-processing script. The front-end web interface is developed using HTML5 and JavaScript. It uses several standard JavaScript libraries including AngularJS and Jquery. The two main objectives of the interface are to provide the user with an easy-to-use and intuitive interface and to break down files into fragments of 1 MB or less, which can be sent over a normal HTTPS connection. The back-end PHP script combines all the file fragments correctly and reassembles the file on the server side.

The user is required to upload a metadata Excel spreadsheet to enable tracking of the sample data, provide the assembler with crucial information about the input and make future sharing of the data possible (See the metadata section). The Excel spreadsheet is read by the front-end web interface, separated into sample entries and converted to JSON files, which are stored in upload directories along with the corresponding sample files. The Batch Upload System then reads the JSON file and parses the information along with the sample WGS files to the BAP. When the metadata Excel spreadsheet has been uploaded and validated by the server and all the WGS files specified in the spreadsheet have been successfully uploaded and assembled, the batch dataset is submitted to the server where the batch upload system is executed. This system handles all batch-related processes, including the execution of the BAP for each of the samples in the batch.

### The metadata

Metadata is a term that covers additional data supporting the main data, which in this case is the WGS sequence data. This could be information about the sampling location, sample date, etc. Proper metadata is a vital part of useful and informative data, especially for epidemiological investigations, because it enables the primary investigator or clinical microbiologist to identify geographical spreading patterns or clusters, leading to the detection of outbreaks.

To ensure a harmonised submission of relevant metadata, a standard template has been developed in Excel spreadsheet format, which is a common data storage tool for users working with WGS data (an example of the a completed metadata sheet can be found in S4 Excelsheet). The metadata template contains 22 attributes of which seven are mandatory. Four of the mandatory attributes are of technical nature and needed by our server for correct execution of the BAP. The list of mandatory metadata is given in Table 1. To use the metadata template for our services, the user has to download the template from the batch upload page, fill out the mandatory fields as a minimum and any optional fields if possible. When the metadata is completed and uploaded, it will be used to ensure that all the necessary WGS files associated with the samples are uploaded before the batch is submitted. All the constrained metadata fields are also validated accordingly, where possible, to ensure that the data provided by the users follow the correct format. All errors encountered are presented to the user through the batch upload interface.

**Table 1:** List of the seven mandatory metadata fields from the CGE metadata template.

Attribute Name	Mandatory	Description
**file_names**	Yes	Name of all files associated to this sample. Multiple filenames should be separated by a space
**pre_assembled**	Yes	Has the uploaded sample data been assembled? yes / no
**sequencing_platform**	Conditional	Choose between: LS454, Illumina, Ion Torrent or ABI SOLiD. Required if the sample is not pre_assembled.
**sequencing_type**	Conditional	Choose between: single, paired or mate-paired. Required if the sample is not pre_assembled.
**country**	Yes	The country from which the sample was collected
**isolation_source**	Yes	Choose between: human, water, food, animal, laboratory, other or unknown
**collection_date**	Yes	The date of the sample collection. Use one of the following format: YYYY-MM-DD or YYYY-MM or YYYY

This list was created to be as closely related to the minimum data required by the National Center for Biotechnology Information and the European Nucleotide Archive as possible.

### The User Profile System

To enable the users to access and re-analyse their WGS data, an interactive User Profile System was constructed. New users for the platform have to create a personal profile consisting of a username, an email and a password. No username or email address can be registered more than once, and only one session can be active per profile at any time. In the user settings menu, the user can change his or her personal information or completely delete the user profile and all user-associated data including metadata and any files the user has uploaded. Data submitted to the server is only accessible to the user account from which it was submitted, unless the data is made publicly available.

### The Sample Management System

**Fig 3:**
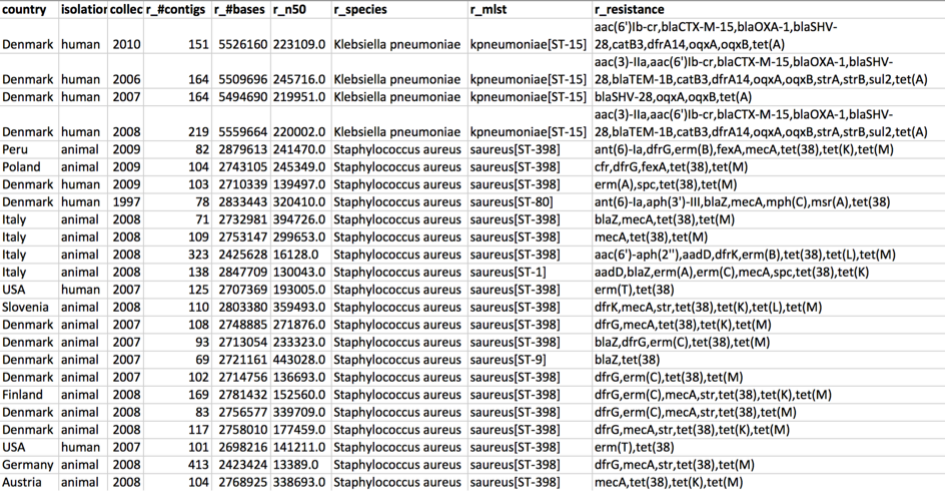
Snippet from the result data for the MLST dataset. The list shows a small selection of the metadata associated with each sample. The column headers are marked in bold and the headers for the services' result are marked with the 'r_' prefix.

When a user submits batch jobs to the server, the results are computed and stored on the server. To access the result of the services, the user can use the Sample Management System (SMS). In the SMS, the user can access information for all submitted samples by the user and the status for all the services. Each service will provide a web link to the results of the service for the given sample, and the sample entry also provides a link to the summary page from the BAP. The user can also edit the metadata for a sample or delete all data for a given sample completely. A deletion of the sample data will delete all stored files for the sample including any service results, and any database entries associated with the sample will also be deleted. In addition, all the service-related results can be deleted individually. The SMS gives the user full power over his or her sample data and removes the need to upload WGS files multiple times to run different services. Using the SMS, the user will be able to reuse his or her uploaded data for any analysis in any available service on the platform. Additionally, the results will also be stored for later viewing and retrieval of the result data, and all the results and metadata for all samples can be downloaded as an Excel spreadsheet for easy downstream analysis. See Fig 3 for a snippet of the result Excel spreadsheet.

### The database structure

The database has two parts: a file storage and a MySQL database. In the file storage, all files associated with a sample are stored within a single directory, and all service-run data is stored in a service directory within the sample directory. This simple structure makes it easy to clean up when the user requests a deletion of a sample or similar.

The MySQL database, in addition to the user login details, stores all the sample metadata and relevant results from the BAP. The MySQL database's most important task is to keep track of files and directories, and any associations between users, samples, files etc. The database makes it possible to quickly retrieve the user’s data so it can be presented to the user. It also enables restriction of data access to authorised users.

### Using the integrated platform

Usage of this platform requires the user to be logged in to a user account. The platform only accepts DNA sequences as input, provided as either fasta or fastq files. The platform provides several results in different formats. Each service creates their own output webpage, and most services also provide files that can be downloaded. For instance, the Assembler provides the contigs as a downloadable file. In addition to the individual service results, the platform also provides a BAP result summary page and some Excel spreadsheets that summarise the results from the analyses. For more details see the usage example in S1 Appendix.

## Results and Discussion

The individual servers included in the pipeline have so far (checked 14 Apr 2016) been tested by users from more than 10,600 locations (unique IP addresses), submitting more than 260,000 jobs. The integrated platform is a response to many user requests for a service that can fulfil their high throughput needs and provide an easier and more intuitive interaction with uploaded WGS data. The data stored on the CGE machines are backed up daily, and can be recovered in case we experience critical server breakdowns involving data loss. The platform has so far met most of the needs from our users, who have provided feedback on their usage of our platform, and we will continue to develop new features and tools to provide an even more powerful platform in the future.

### Time consumption

To test the time consumption of the services in the pipeline and the pipeline it self, we sent 476 runs from the European Nucleotide Archive through the pipeline. The runs contained Illumina paired-end fastq files. The pipeline it self took about 19–28 minutes per run on average, where the majority of the time was spent on the assembly part. For the individual services: the Assembler took around 10–20 minutes on average, but scales with the sequencing depth (~5 s/depth). KmerFinder took 5–10 minutes on average, and also scales with sequencing depth (~2s/depth). For the remaining services, the average time was: ResFinder 3–4 minutes, MLST 6–7 minutes, PlasmidFinder 1–2 minutes and pMLST 1–3 minutes.

Genome assembly quality can vary a lot depending on the species, but also on the sequencing depth. As a rule of thumb (based on N50), most cases will produce good assemblies with a depth above 50, though for some species like *Salmonella*, the N50 seems to increase up to a depth around 100 before it stagnates.

This estimation was based on the results from the above-mentioned runs, and a plot of the data can be found in S2 Appendix.

When a user submits multiple samples in one batch, the sample analyses are executed in serial. The total time of the whole batch analysis will thus be a cumulative sum of all the analyses.

### Platform Benchmark

**Fig 4:**
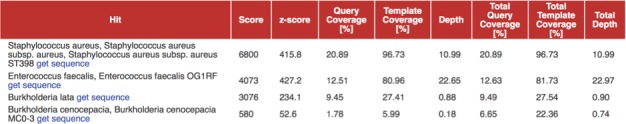
KmerFinder top results for sample *S*. *aureus* F38. The result shows that there are more than just significant hits for *S*. *aureus*. This indicates that the sample was contaminated and thus not a single isolate.

The concordance to previously obtained results was very high. The Assembler was able to assemble all samples, both Illumina and Ion Torrent fastq files, and the contigs had an average N50 of 220,000 bases. A few samples had low N50, which in combination with a high number of contigs, likely indicates poor sequencing quality rather than problems with the assembler (see Table 2). Also, two of the samples (*S*. *aureus* ST20091526 and ST20101526) had to be reassembled with the trimming option enabled to produce contigs. In the case of *S*. *aureus* F38, the sample had 959 contigs and a genome size of 5,721,289 bases, which is about twice the norm for *S*. *aureus*. Further investigation of the KmerFinder results of this sample revealed that the sample probably was contaminated with *E*. *faecalis* (see Fig 4). Even with the contamination, the platform was still able to predict the MLST of the *S*. *aureus* in the sample. Overall the Assembler is good at assembling draft genomes for both the Illumina data in the MLST dataset and the Ion Torrent data in the VirulenceFinder dataset.

**Table 2:** Assembly by the internal Assembler.

Species		No. Contigs	No. Bases	N50
*Klebsiella pneumoniae*	**Min**	151	5494690	219951
	**Median**	164	5517928	221556
	**Mean**	175	5522553	227195
	**Max**	219	5559664	245716
	**SD**	30	27880	12436
*Staphylococcus aureus*	**Min**	57	2234148	7764
	**Median**	104	2760734	207528
	**Mean**	149	2765851	216002
	**Max**	959	5721289	685832
	**SD**	137	342548	140720
*Escherichia coli*	**Min**	52	4055781	21906
	**Median**	247	5245766	94653
	**Mean**	267	5158599	88163
	**Max**	647	5523711	176604
	**SD**	121	269720	35535

The table provides a summary of the annotation for the assemblies of the samples in the MLST and the VirulenceFinder dataset. The annotations metrics compared in this benchmark are: Number of contigs, number of bases and the N50.

For all 335 dataset of all six species the KmerFinder predicted the species exactly as it should (see Table 3). The MLST previously predicted was also found in all but three samples where the MLST could not be predicted due to problems with the assembled contigs, where one of the genes were not assembled properly (see Table 4). The same was observed for the prediction of resistance genes (see Table 5) and virulence genes (see Table 6). Here the pipeline found the vast majority of the genes (93% and 98% respectively), but some were missed due to the genes being split on two contigs. For PlasmidFinder, the benchmark only showed one plasmid disagreeing with the published manuscript (see Table 7), and the pMLST benchmark shows the exact same results as previously published (see Table 8).

**Table 3:** Species prediction by KmerFinder.

Species	Concordance	Discordance	Unknown	Total
*Escherichia coli* (VF)	42	0	1	43
*Escherichia coli* (RF)	49	0	0	49
*Salmonella enterica*	50	0	0	50
*Enterococcus faecalis*	50	0	0	50
*Enterococcus faecium*	45	1	0	46
*Klebsiella pneumoniae*	4	0	0	4
*Staphylococcus aureus*	93	0	0	93
**Total**	**333**	**1**	**1**	**335**

All species predictions are as they should be. Two samples were predicted differently than expected. The first was a sample from the ResFinder dataset expected to be E. faecium, but predicted to be Enterococcus hirae. The sample was proven to be E. hirae in the original manuscript[8]. The second sample was from the VirulenceFinder dataset, this sample was expected to be E. coli, but the prediction gave no significant result. This means that no significant amount of k-mers from the sample matched anything in the KmerFinder database. The original manuscript also disproved this sample as an E. coli[5].

**Table 4:** Sequence type prediction by the MLST algorithm.

Species	Concordance	Discordance	Unpredicted	Total
*Klebsiella pneumoniae*	4	0	0	4
*Staphylococcus aureus*	90	0	3	93
**Total**	**94**	**0**	**3**	**97**

This table summarises the results from the MLST. There is concordance when the MLST is predicted to be the same as the expected, and discordance when they differ. If the MLST has not been provided with the metadata, it is categorised as unknown, and if the MLST algorithm is not able to predict the MLST, it is categorised as unpredicted. Nearly all samples are predicted correctly, only three samples' ST were not identified. By investigating their genes independently, we find that the three *S*. *aureus* samples M-5, T5 and 7413532–2 all belongs to clonal complex 398; the missing ST was due to a single gene being badly assembled. Similarly MLST for *S*. *aureus* 55488_TG8086 did not provide a ST due to bad assembly of a gene, but reassembly with the trimming option, enabled MLST to identify the ST.

**Table 5:** Resistance gene prediction by the ResFinder.

Species	Published	Concordance	Missing	Split-miss	Extra 80%	Vary
*Escherichia coli*	112	109	0	2	0	1
*Enterococcus faecium*	139	132	0	6	7	1
*Enterococcus faecalis*	221	189	1	31	14	0
*Salmonella enterica*	128	126	0	1	6	1
**Total**	**600**	**556**	**1**	**40**	**27**	**3**

This table summarises the resistance gene prediction results. The dataset used is the same as in the ResFinder manuscript. It is worth noting that ResFinder finds 93% of the genes automatically. Of the last 7% of the genes around 6.7% are not found due to bad assemblies, where the genes are either truncated or split on two contigs. To find these split genes, the user will have to do further investigations, as was done in the ResFinder manuscript. 3 of the 600 genes expected were predicted to be another variant of the same gene, which could be due to changes made in the ResFinder database since the manuscript was published. Only 1 of the expected genes could not be identified even when rerunning ResFinder using a threshold of 80% and a minimum length of 40%, though with this low threshold ResFinder also find 27 extra genes that were not published in the ResFinder manuscript.

**Table 6:** Virulence gene prediction by the VirulenceFinder.

Species	Published	Concordance	Missing	Split-miss
*Escherichia coli*	106	104	1	1
**Total**	**106**	**104**	**1**	**1**

This table summarises the result from the virulence gene identification benchmark. The dataset used here is the same as in the VirulenceFinder manuscript [5]. The table shows how many genes were observed using the platform and compares it to how many genes were found in the previous publication. 98% of the genes are identified by the platform automatically. Of the two unidentified genes, one could not be identified even with lower thresholds; the other however, was found by additional investigations to be split in two parts placed on separate contigs. This was also the conclusion in the VirulenceFinder manuscript [5].

**Table 7:** Plasmid prediction by the PlasmidFinder algorithm.

Species	Published	Concordance	Missing	Extra
*Salmonella enterica*	104	103	1	7
**Total**	**104**	**103**	**1**	**7**

The results from the PlasmidFinder benchmark are summarised. The dataset used here are the same as in the PlasmidFinder manuscript. The table shows how many plasmids were observed using the platform and compares it to how many plasmids were found in the previous publication. 99% of the published plasmids are identified by the platform automatically, but the pipeline also detected 7 plasmids that were not previously published. The extra plasmids are found since the original study used a threshold of 90%, where as the recommended threshold from the authors and also the threshold used in the pipeline is 80% identity.

**Table 8:** Plasmid sequence type prediction by the pMLST algorithm.

Species	Concordance	Discordance	Total
*Salmonella enterica*	39	1	40
**Total**	**39**	**1**	**40**

This table summarises the result from the pMLST benchmark. The dataset used here is the same as in the pMLST manuscript. The table shows how the concordance between the predicted pMLSTs and the previously published pMLSTs. 39/40 pMLST predictions agreed with the published pMLSTs, the discordant pMLST was caused by a later change of ST id for that gene, and the predicted pMLST alleles are actually the same as previously published.

For complete dataset details and results see S1 Excelsheet, S2 Excelsheet and S3 Excelsheet.

### Justification of service choices

The initial selection of services integrated in the platform was chosen because they individually have been benchmarked to have a high accuracy, and since they have a similar output format and setup making their integration into the platform easy. Each service has its limitations and weaknesses, and as can be seen in the benchmark, our services are no different. Bad assemblies resulting from bad sequencing will impact the performance of BLAST-based methods such as ResFinder and VirulenceFinder, since genes might be truncated and/or divided on multiple contigs, or missed if the genes cannot be assembled at all. As shown in the benchmark, the MLST algorithm, which is very sensitive to bad assemblies, actually predicts nearly all MLST types. This indicates that the Assembler used is appropriate for assembling the data tested. If the user has special requirements for different assembly methods, the platform also allows them to do the assembly locally previous to submission and upload the resulting genome contigs to the platform.

### Intended purpose and plans

The platform described here provides a single analytic framework for multiple analyses from batch upload of entire runs with multiple single isolates, where the use of the platform does not require the user to possess any advanced prior knowledge on bioinformatics. The BAP automatically decides on the appropriate scheme for e.g. MLST typing based on the species identification and provides a short printable report for each single entry. In addition, the SMS provides an interface to all previously uploaded samples and can as such be used as a repository, though we do not offer infinite storage of non-public data. The intended users would be people performing WGS for human or veterinary diagnostics or surveillance without any advanced knowledge of bioinformatics. The service is web-based and free of charge, and is thus also available to the developing world.

It is our intention to further improve the platform as other services and features become available. Examples of such services are serotyping of *E*. *coli* and *Salmonella* [24,25] cluster analysis using multiple samples (e.g. phylogenetic trees based on single nucleotide polymorphisms), integration of geolocation for samples and results into the SMS in a map/list web interface and implementing a sharing system so the users can select a group of other users that should have access to subsets of the data. In addition, an optional automatic upload to the European Nucleotide Archive (ENA) of sample metadata and WGS data will soon be implemented.

### Privacy Statement

At CGE we respect user privacy, and we will as such not use any data uploaded by our users or generated by our services for anything unless the user explicitly have marked his or her samples as public available to all. The user can at all time delete his or her samples and all data related them from the SMS interface.

## Conclusions

A combined bioinformatics platform was developed and made publicly available, providing batch upload, automated and simultaneous analysis for bacterial species, subtype, antimicrobial resistance and selected virulence genes and plasmids, the ability to reuse uploaded data, and data safety. The platform was benchmarked and deemed to be very fast and accurate, and may be of immediate relevance as a guide in combination with epidemiological information and other conventional methods for investigators using WGS for clinical diagnostic, research and surveillance.

## Supporting Information

S1 Appendix Word document containing a usage example of the CGE platform. (DOCX)

S2 Appendix Word document containing a graph showing N50 as a function of depth. (DOCX)

S1 Excelsheet MLST dataset: metadata and analysis results. (XLSX)

S2 Excelsheet ResFinder dataset: metadata and analysis results. (XLSX)

S3 Excelsheet VirulenceFinder dataset: metadata and analysis results. (XLSX)

S4 Excelsheet Example of a batch-upload prepared metadata sheet. (XLSX)
