# Supplementary material for: A Bacterial Analysis Platform: An Integrated System for Analysing Bacterial Whole Genome Sequencing Data for Clinical Diagnostics and Surveillance
Source: PLoS One. 2016 Jun 21;11(6):e0157718. doi: 10.1371/journal.pone.0157718 (PMC4915688; doi:10.1371/journal.pone.0157718)
Supplement: S1 Appendix — (DOCX) [file pone.0157718.s001.docx]

*Appendix 1 - Usage example*

The Platform is, as previously mentioned, intended to be fairly easy and straightforward to use for non-bioinformaticians. First step is to create an account. This can be done from the service overview page: https://cge.cbs.dtu.dk/services/, by clicking on the new button in the top right corner. The user selects a username and password, provide an email address, fill out the Captcha code, and press 'Create User'. Then the user needs to wait for the confirmation email and follow the activation link. Now the user is logged in, and can use his or hers username and password to log into the new account in the future.

To upload sequencing data the user goes to the batch-upload interface. This can be accessed by activating the user menu through hovering over the user name in the top right corner, and selecting "Batch Uploader", causing the batch-upload interface to be shown. To use this interface the user follows the 5-step guide explained on the page. First the metadata template must be downloaded. Secondly, the mandatory metadata and all the other metadata fields, which are appropriate, are filled out.

For this example let us download an *Escherichia coli* reference from NCBI (http://www.ncbi.nlm.nih.gov/genome/167?genome_assembly_id=161521). Since this is just a test example, we can just fill out the metadata as we see fit: sample_name is "MG1655", file_names is "GCF_000005845.2_ASM584v2_genomic.fna.gz", sequencing_platform, sequencing_type and pathogenic are "unknown", pre_assembled is "yes", organism is "*Escherichia coli*", country is "USA", isolation_source is "human", and collection_date is "1922". Third step is to upload the completed metadata sheet, and since we filled it out correctly, we get no errors. Fourth step is to select the files. Here we select the " GCF_000005845.2_ASM584v2_genomic.fna.gz" which we downloaded from NCBI. Fifth step is to click submit, and now we just have to wait for the result. We can either wait for the page to redirect us to a summary report of the uploaded sample, add our email, and get notified when the job has finished, or go directly to the sample manager to follow the progress of the job live. Let us open a new window tab and with the main service page, and select "Sample Manager" from the user menu. In the sample manager, we see a table containing one entry named MG1655, which was the sample_name we provided. To the left of the name we see a green plus sign, which when clicked shows a link and a status for each of the services being run on that given sample. By clicking the link on a service where the status is "Success", we can see the result of that service. When all the services have finished, we are also able to click on the MG1655 link and see the BAP summary page for the uploaded sample. As expected the sample is predicted to be *Escherichia coli*, and we actually find the exact match since this reference genome is present in the KmerFinder database. Going back to the sample manager page, we note that there is a link to download all data in an Excel spreadsheet. In the spreadsheet we find our sample and can see all the metadata that we provided in addition to a summary of the results. All result field names have a "r_" prefix.
