# Supplementary material for: A Bacterial Analysis Platform: An Integrated System for Analysing Bacterial Whole Genome Sequencing Data for Clinical Diagnostics and Surveillance
Source: PLoS One. 2016 Jun 21;11(6):e0157718. doi: 10.1371/journal.pone.0157718 (PMC4915688; doi:10.1371/journal.pone.0157718)
Supplement: S2 Appendix — (DOCX) [file pone.0157718.s002.docx]

**Graph: N50 as a function of depth**


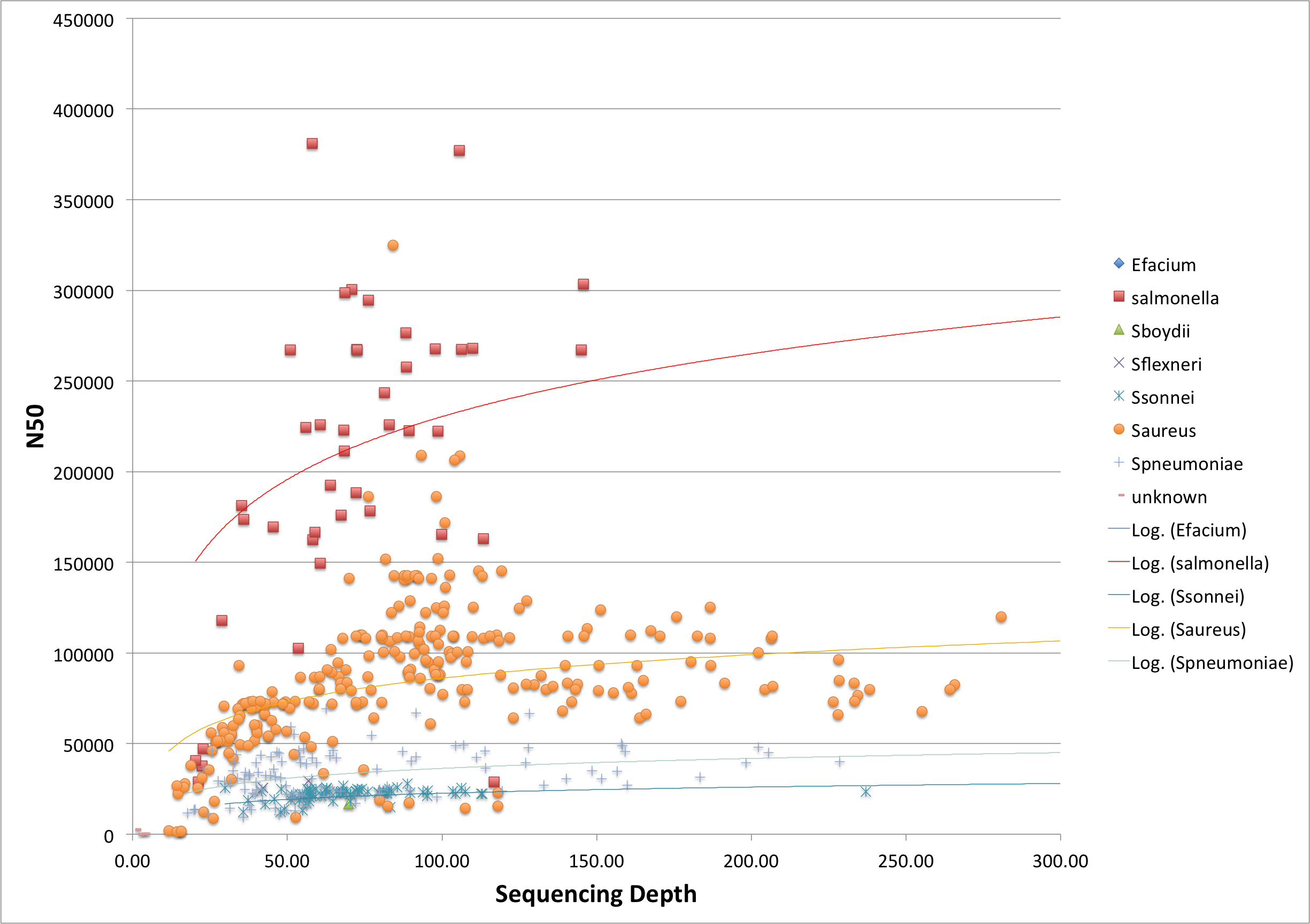


**This graph show the relationship between N50 and depth for each species in the dataset.**

The x-axis was constrained to 300 max, which excluded all the *E. faecium* data, where all depth in the dataset were above 300. Since all the *E. faecium* data (not shown) have an N50 of around 50000, we are not able to estimate when the stagnation is reached.

**Method**

Downloaded all the files for each run (see list below), ran them through the CGE Batchupload and Pipeline, and then plottet the N50 as a function of depth for each species in the dataset in Excel.

**The following list of 537 runs were used to create the above graph:**

ERR016635

ERR016639

ERR016640

ERR016641

ERR016645

ERR016646

ERR016647

ERR016648

ERR016649

ERR016650

ERR016651

ERR016652

ERR016653

ERR016654

ERR016655

ERR016656

ERR016658

ERR016659

ERR016660

ERR016661

ERR016662

ERR016663

ERR016664

ERR016665

ERR016666

ERR016667

ERR016668

ERR016670

ERR016671

ERR016672

ERR016673

ERR016676

ERR016677

ERR016678

ERR016679

ERR016681

ERR016682

ERR016684

ERR016685

ERR016687

ERR016689

ERR016690

ERR016692

ERR016693

ERR016694

ERR016695

ERR016696

ERR016697

ERR016698

ERR016699

ERR016700

ERR016701

ERR016702

ERR016703

ERR016705

ERR016706

ERR016707

ERR016708

ERR016709

ERR016710

ERR016711

ERR016712

ERR016714

ERR016716

ERR016719

ERR016720

ERR016721

ERR016724

ERR016725

ERR016726

ERR016727

ERR016728

ERR016729

ERR016731

ERR016732

ERR016734

ERR016735

ERR016736

ERR016737

ERR016738

ERR016739

ERR016740

ERR016741

ERR016742

ERR016743

ERR016744

ERR016745

ERR016747

ERR016750

ERR016751

ERR016752

ERR016754

ERR016757

ERR016758

ERR016761

ERR016765

ERR016766

ERR016771

ERR016774

ERR016777

ERR016782

ERR016793

ERR016794

ERR016800

ERR016802

ERR016813

ERR016823

ERR016825

ERR016826

ERR016829

ERR016830

ERR016831

ERR016832

ERR017168

ERR017169

ERR017170

ERR017171

ERR017172

ERR017173

ERR017174

ERR017175

ERR017176

ERR017177

ERR017178

ERR017179

ERR017180

ERR017181

ERR017182

ERR017183

ERR017184

ERR017185

ERR017186

ERR017187

ERR017188

ERR017189

ERR017190

ERR017191

ERR017192

ERR017193

ERR017194

ERR017195

ERR017196

ERR017197

ERR017198

ERR017199

ERR017200

ERR017201

ERR017202

ERR017203

ERR017204

ERR017205

ERR017206

ERR017207

ERR017208

ERR017209

ERR017210

ERR017211

ERR017212

ERR017213

ERR017214

ERR017215

ERR017216

ERR017217

ERR017218

ERR017219

ERR017220

ERR017221

ERR017222

ERR017223

ERR017224

ERR017225

ERR017226

ERR017227

ERR017228

ERR017229

ERR017230

ERR017231

ERR017232

ERR017233

ERR017234

ERR017235

ERR017236

ERR017237

ERR017238

ERR017239

ERR017240

ERR017241

ERR017242

ERR017243

ERR017244

ERR017245

ERR017246

ERR017247

ERR017248

ERR017249

ERR017250

ERR017251

ERR017252

ERR017253

ERR017254

ERR017255

ERR017256

ERR017257

ERR017258

ERR017259

ERR017260

ERR017261

ERR017262

ERR017263

ERR017264

ERR017265

ERR017266

ERR017267

ERR017268

ERR017269

ERR017270

ERR017271

ERR017272

ERR017273

ERR017274

ERR017275

ERR017276

ERR017277

ERR017278

ERR017279

ERR017280

ERR017281

ERR017282

ERR017283

ERR017284

ERR017285

ERR017286

ERR017287

ERR017288

ERR017289

ERR017290

ERR017291

ERR017292

ERR017293

ERR017294

ERR017295

ERR017296

ERR017297

ERR017298

ERR017299

ERR017300

ERR017301

ERR023807

ERR023808

ERR023813

ERR023814

ERR023815

ERR023816

ERR023818

ERR023819

ERR023822

ERR023823

ERR023824

ERR023825

ERR023826

ERR023827

ERR023828

ERR023829

ERR023835

ERR023836

ERR023837

ERR023838

ERR024064

ERR024084

ERR024092

ERR024377

ERR024387

ERR024388

ERR024390

ERR024391

ERR024392

ERR024394

ERR024395

ERR024396

ERR024397

ERR024398

ERR024400

ERR024401

ERR024402

ERR024404

ERR024405

ERR024406

ERR024407

ERR024408

ERR024409

ERR024410

ERR024411

ERR024413

ERR024417

ERR024604

ERR024605

ERR024606

ERR024607

ERR024608

ERR024609

ERR024610

ERR024611

ERR024612

ERR024614

ERR024616

ERR024617

ERR024618

ERR024619

ERR024620

ERR024621

ERR024622

ERR024623

ERR024624

ERR024625

ERR024626

ERR024627

ERR024668

ERR024671

ERR024672

ERR024673

ERR024674

ERR024675

ERR024678

ERR024679

ERR024681

ERR024682

ERR024683

ERR024684

ERR024685

ERR024687

ERR024688

ERR024689

ERR024691

ERR024692

ERR024974

ERR024976

ERR024977

ERR024978

ERR024979

ERR025682

ERR025686

ERR025687

ERR025688

ERR025689

ERR025690

ERR025691

ERR025692

ERR025693

ERR025715

ERR025716

ERR025717

ERR025718

ERR025719

ERR025721

ERR025722

ERR025724

ERR025725

ERR025726

ERR025727

ERR025729

ERR025730

ERR025731

ERR025732

ERR025735

ERR025737

ERR025738

ERR025739

ERR025741

ERR025742

ERR025743

ERR025744

ERR025746

ERR025747

ERR025748

ERR025749

ERR025750

ERR025751

ERR025752

ERR025753

ERR025754

ERR025755

ERR025756

ERR025758

ERR025759

ERR025761

ERR025762

ERR025763

ERR025764

ERR025765

ERR025766

ERR025767

ERR025768

ERR026003

ERR026007

ERR026008

ERR026009

ERR026010

ERR026011

ERR026012

ERR026649

ERR026650

ERR026651

ERR026652

ERR026653

ERR026655

ERR026656

ERR026658

ERR026662

ERR026663

ERR026664

ERR026665

ERR026666

ERR026667

ERR026668

ERR026669

ERR026674

ERR026675

ERR026676

ERR026677

ERR026678

ERR026679

ERR026680

ERR026683

ERR026684

ERR026687

ERR026688

ERR026690

ERR026691

ERR026692

ERR026694

ERR026695

ERR026697

ERR026701

ERR026703

ERR026705

ERR026706

ERR026707

ERR026708

ERR026709

ERR026710

ERR064898

ERR064899

ERR064900

ERR064901

ERR064902

ERR064903

ERR064904

ERR064906

ERR064907

ERR064908

ERR064909

ERR064910

ERR064911

ERR064912

ERR064914

ERR064915

ERR064916

ERR064917

ERR064918

ERR064919

ERR064920

ERR064921

ERR064922

ERR064923

ERR064924

ERR064925

ERR064926

ERR064928

ERR064929

ERR064930

ERR064931

ERR064932

ERR064933

ERR064934

ERR064935

ERR064937

ERR064938

ERR064939

ERR064940

ERR064941

ERR064942

ERR064943

ERR064944

ERR064945

ERR064946

ERR064947

ERR064948

ERR064949

ERR064950

ERR064951

ERR064953

ERR064954

ERR064955

ERR064956

ERR064958

ERR064959

SRR980548

SRR980549

SRR980550

SRR980551

SRR980552

SRR980553

SRR980554

SRR980555

SRR980556

SRR980557

SRR980558

SRR980559

SRR980560

SRR980561

SRR980562

SRR980563

SRR980564

SRR980565

SRR980566

SRR980567

SRR980568

SRR980569

SRR980570

SRR980571

SRR980572

SRR980573

SRR980574

SRR980575

SRR980576

SRR980577

SRR980578

SRR980579

SRR980580

SRR980581

SRR980582

SRR980583

SRR980584

SRR980585

SRR980586

SRR980587

SRR980588
